# Supplementary material for: Genetic comparisons of interleukin-17 reveal a framework for complex signaling evolution
Source: bioRxiv. 2026 Apr 14:2026.04.13.718218. Preprint. [Version 1] doi: 10.64898/2026.04.13.718218 (PMC13105070; doi:10.64898/2026.04.13.718218)
Supplement: Supplement 3 [file media-3.zip › Supplemental files for paper/ERC/IL17xIL17_comparisons/IL17RE_IL25.pdf]

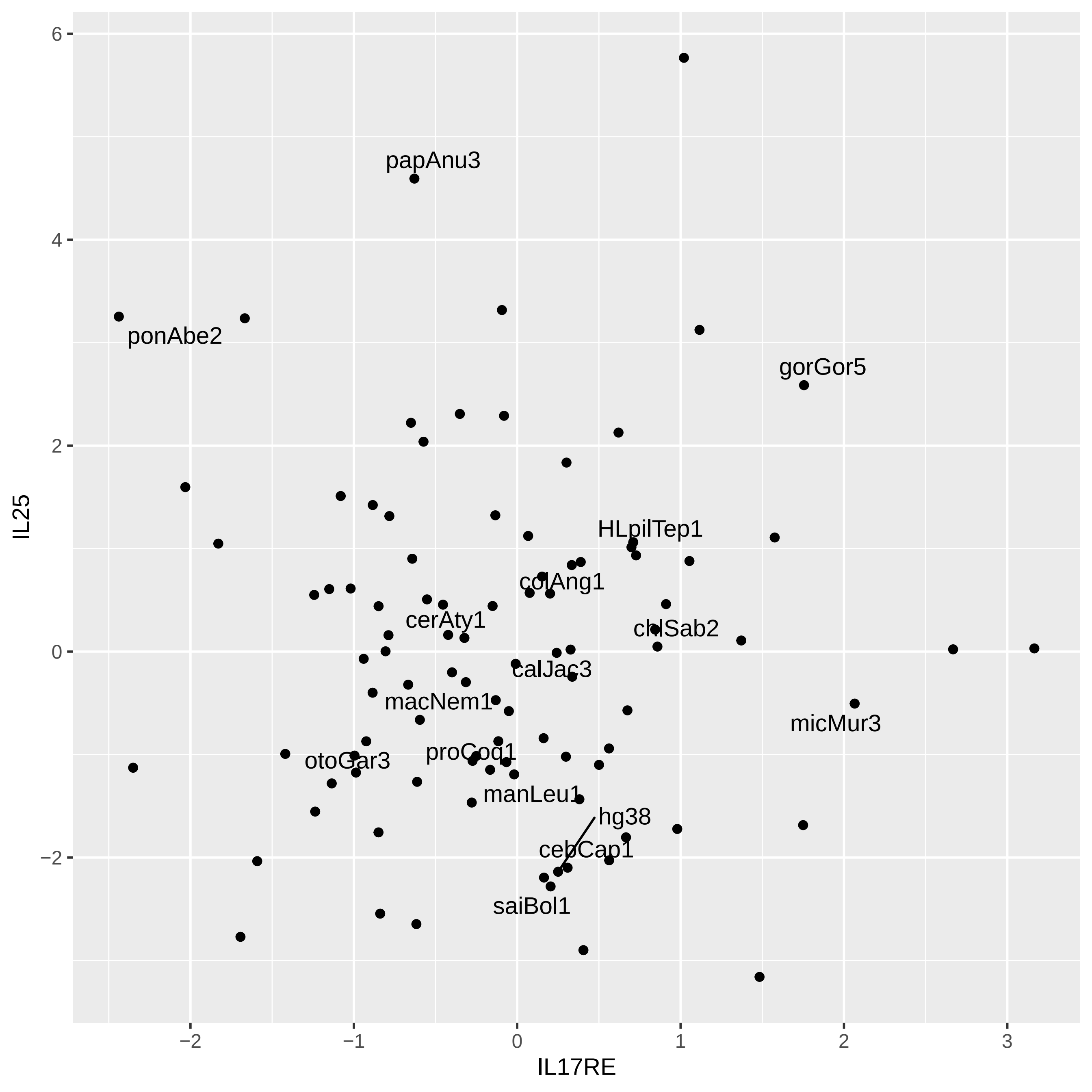

papAnu3
ponAbe2
gorGor5
HLpilTep1
colAng1
cerAty1
chlSab2
calJac3
macNem1
micMur3
proCoq1
otoGar3
manLeu1
hg38
cebCap1
saiBol1
IL25
IL17RE
6
4
2
0
−2
−2
−1
0
1
2
3
